# Supplementary figures and images for: Preparation and characterization of monoclonal antibodies recognizing two CD4 isotypes of Microminipigs
Source: PLoS One. 2020 Nov 25;15(11):e0242572. doi: 10.1371/journal.pone.0242572 (PMC7688132; doi:10.1371/journal.pone.0242572)

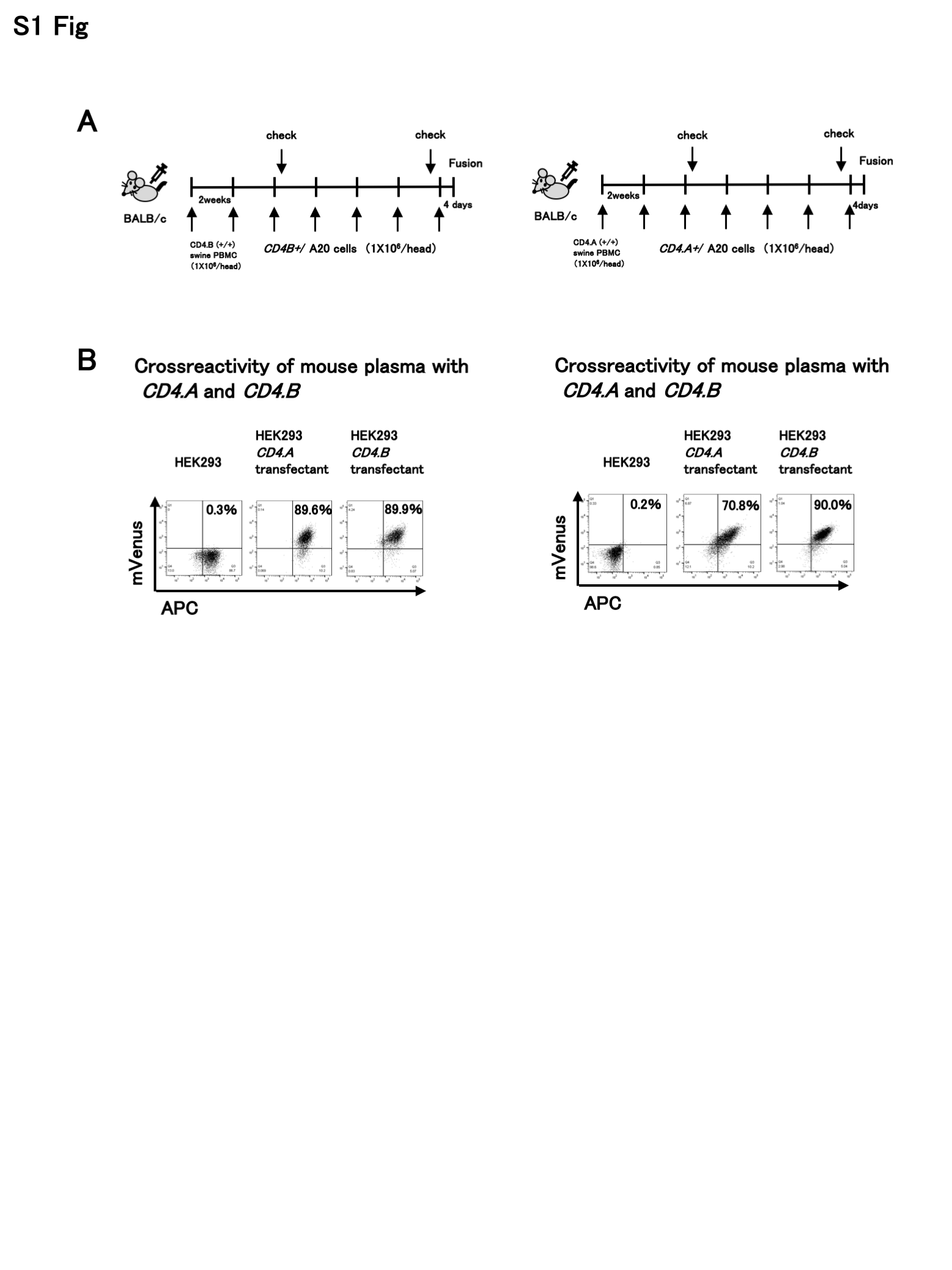

Supplement: S1 Fig — A. Protocol for immunization of the BALB/c mice with swine CD4 antigen. Swine PBMC or CD4 isotype transfectants (CD4A+/A20 or CD4B+/A20) were immunized biweekly. One week after 3rd and 6th immunization, peripheral blood (PB) was collected and antibody titers were examined. B. Antibody reactivity to CD4.A and CD4.B was checked by FCM. The fluorescent intensity of mVenus shows the transfection efficiency. Mouse antisera were used for the first antibody for staining the cells. The numbers shown in the panels are the percentage of the positive cells. Left panels show the CD4.B specific mAb preparation. Right panels show the CD4.A and CD4.B specific mAb preparation. (TIF) [file pone.0242572.s001.tif]

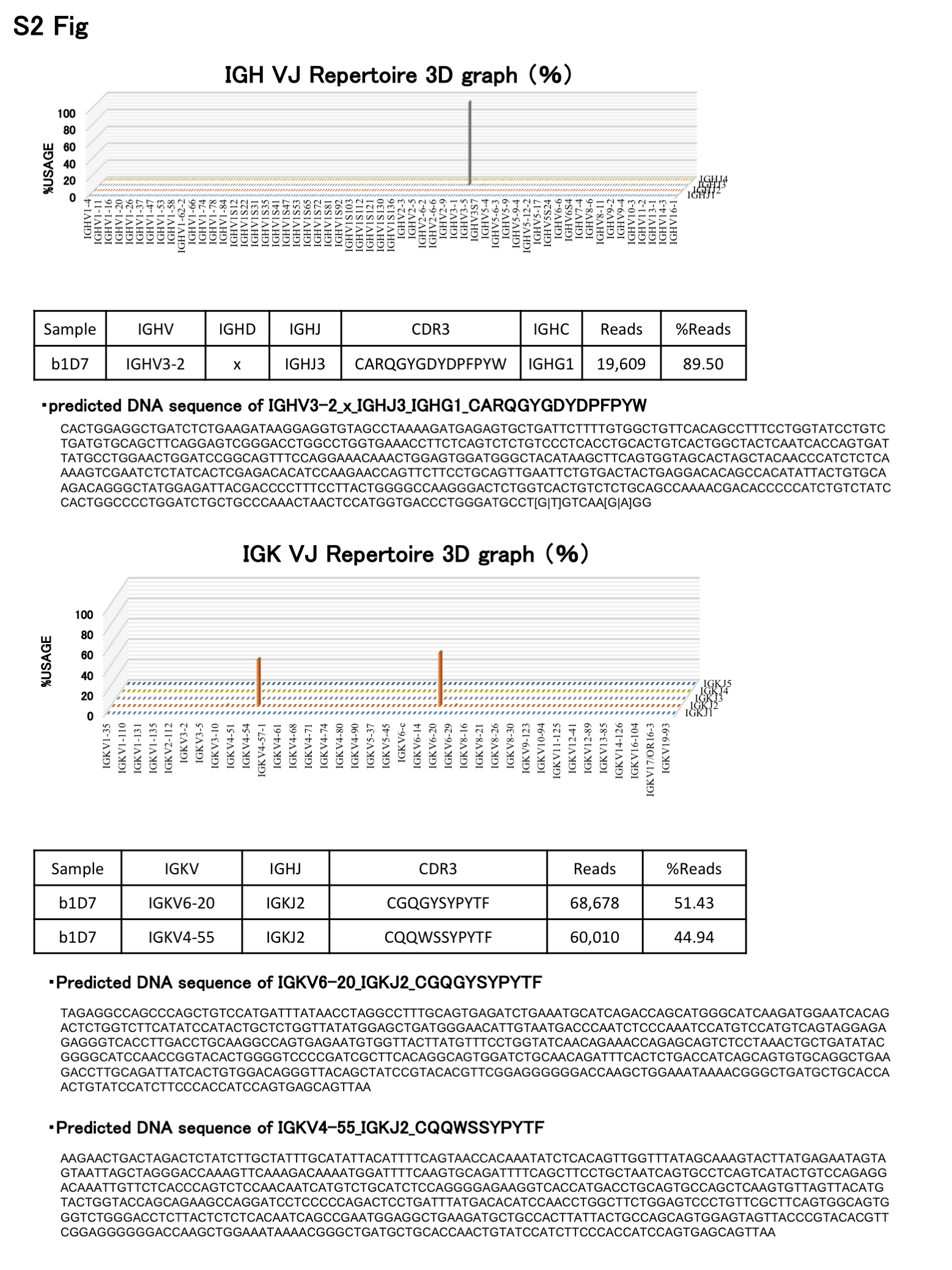

Supplement: S2 Fig — The repertoire analysis was performed by Repertoire Genesis (Osaka, Japan). The types of heavy chain and light chains and the sequences are shown. (TIF) [file pone.0242572.s002.tif]

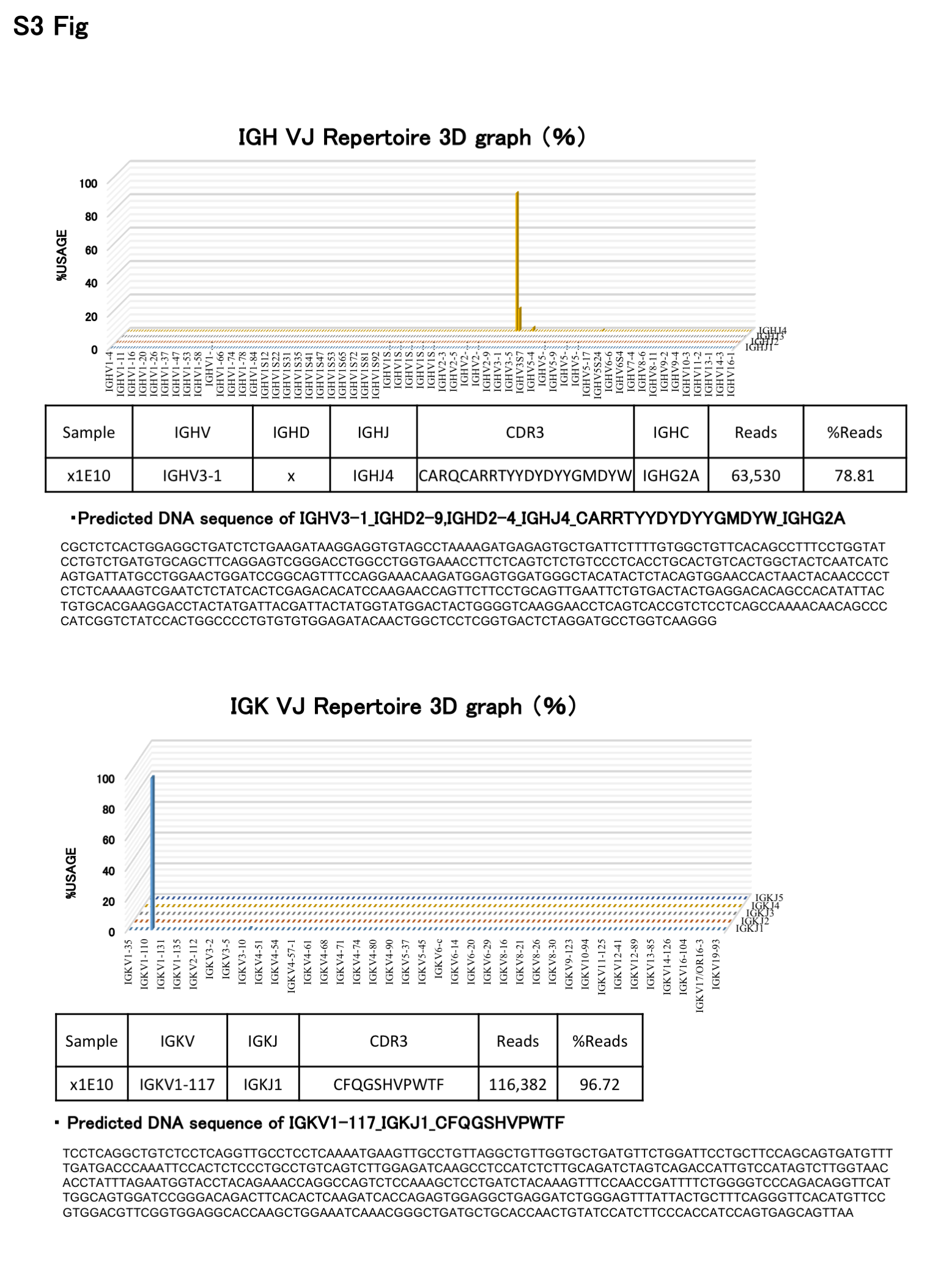

Supplement: S3 Fig — The repertoire analysis was performed by Repertoire Genesis (Osaka, Japan). The type of heavy chain and light chains and the sequences are shown. (TIF) [file pone.0242572.s003.tif]

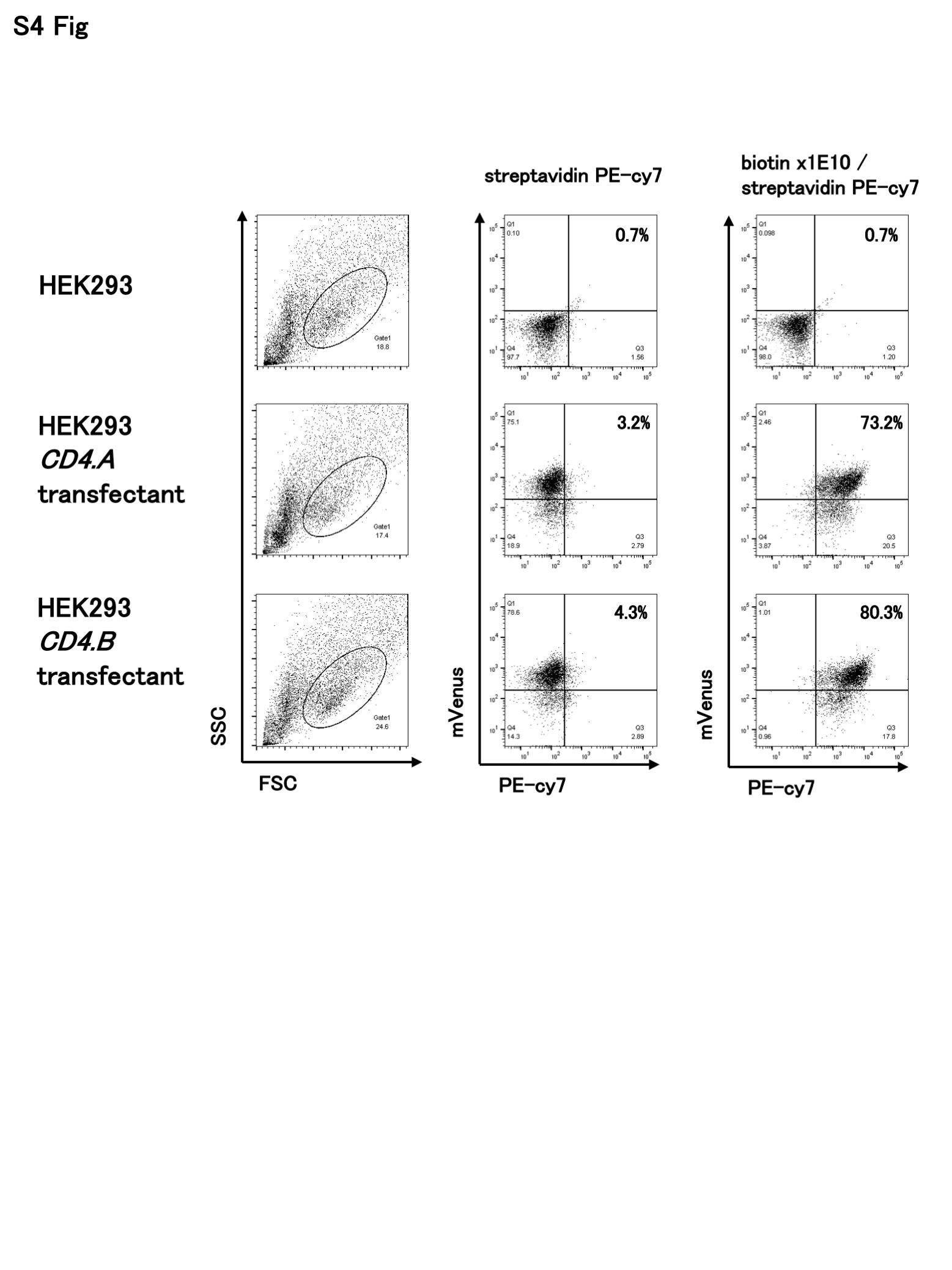

Supplement: S4 Fig — The x1E10 mAb was labeled with biotin and HEK293 transfected with CD4.A or CD4.B gene was stained with biotinylated x1E10 and streptavidin PE-cy7. The reactivity was evaluated by FCM showing that the reactivity of labeled antibody against both alleles is almost the same. (TIF) [file pone.0242572.s004.tif]

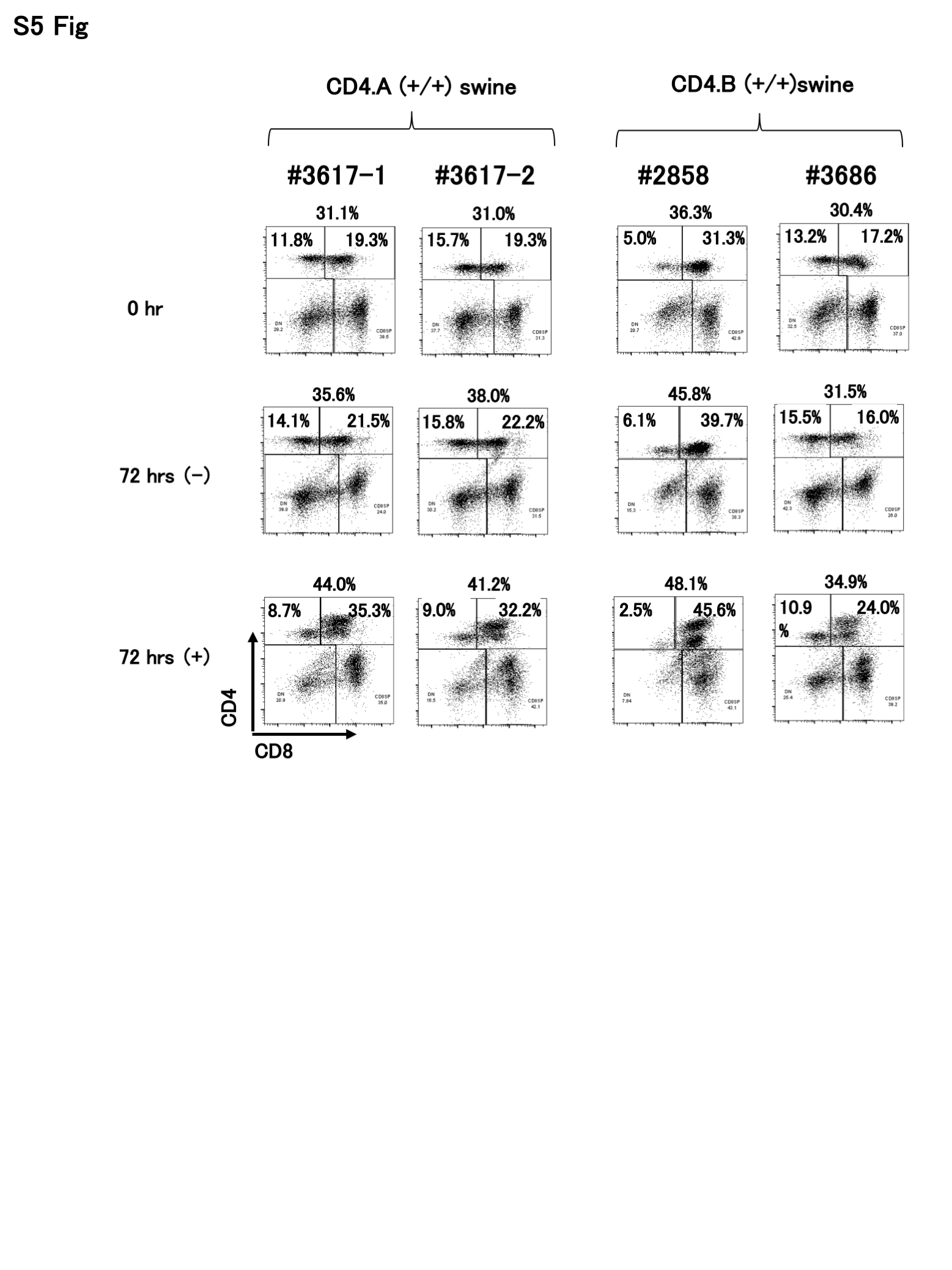

Supplement: S5 Fig — Swine PBMCs (CD4.A(+/+) and CD4.B(+/+)) were stimulated and stained with the mAb (x1E10) followed by anti-mouse IgG-PE, and anti-CD8 mAb and analyzed by FCM as mentioned in Fig 5. Left panels; CD4.A (+/+) swine. The same swine collected at a different time (#3617–1 and #3617–2). Right panels; CD4.B (+/+) swine. Two different swine (#2858, #3686) are shown. The ratio of CD4SP and DP cells in the lymphocyte gated cells are shown in the panels. The number shown above each panel represents the sum of CD4SP and DP cell percentages. (TIF) [file pone.0242572.s005.tif]

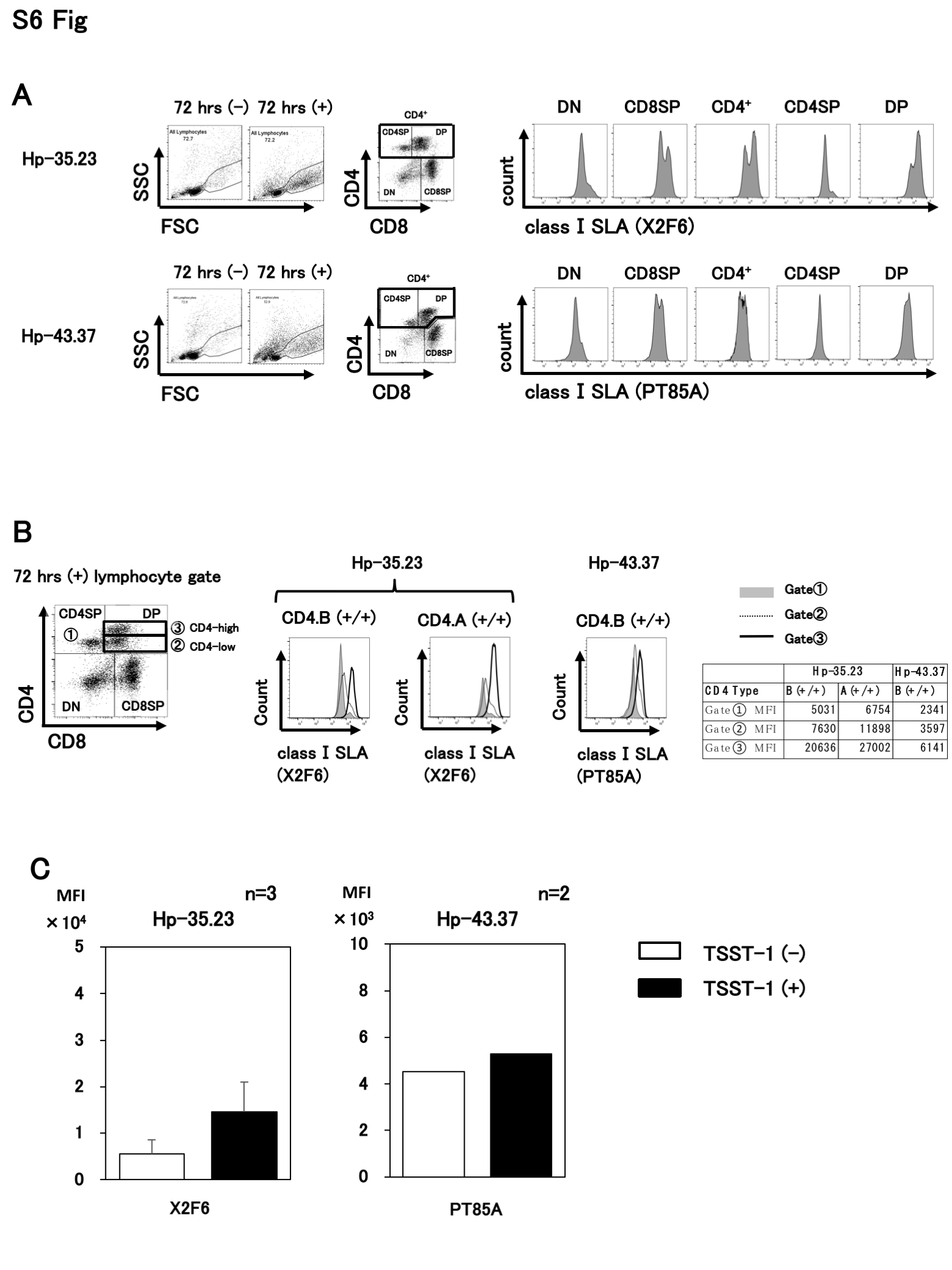

Supplement: S6 Fig — Swine PBMCs were stained with x1E10 and anti-CD8 mAb and analyzed by FCM. A. The left two panels show the representative pattern of FSC/SSC after 72 hrs of culture with/without TSST-1 stimulation. As the large cells are increased by TSST-1 stimulation, a small and large lymphocyte gate was used for the analysis. CD4/CD8 expression is shown in middle panels. Upper panel shows the CD4/CD8 pattern of Hp-35.23 and lower panels; Hp-43.37. Right panels show the histograms of the class I SLA expression of the lymphocyte-gated T cell subsets after TSST-1 stimulation. X2F6 was used for Hp-35.23 and PT85A was used for Hp-43.37. B. The left panel shows the gate of each CD4+ fraction analyzed for the expression of class I SLA. The CD4/CD8 DP cells were divided into CD4 high and CD4 low groups to examine the expression levels of class I SLA and the MFI data are shown in the right table. The middle panels show the overlay pattern of class I SLA expression in each group of Hp-35.23 and Hp-43.37 swine. The groups were further divided into CD4.A(+/+) and CD4.B(+/+) groups that are shown in the panels. C. Class I SLA expression on T cells after TSST-1 stimulation. Left panel; The MFIs of class I SLA expression on DP T cells of Hp-35.23 swine with/without stimulation of TSST-1. Right panel; class I SLA expression on DP T cells of Hp-43.37. Open bars; 72 hrs culture without TSST-1, Black bars; 72 hrs culture with TSST-1. (TIF) [file pone.0242572.s006.tif]

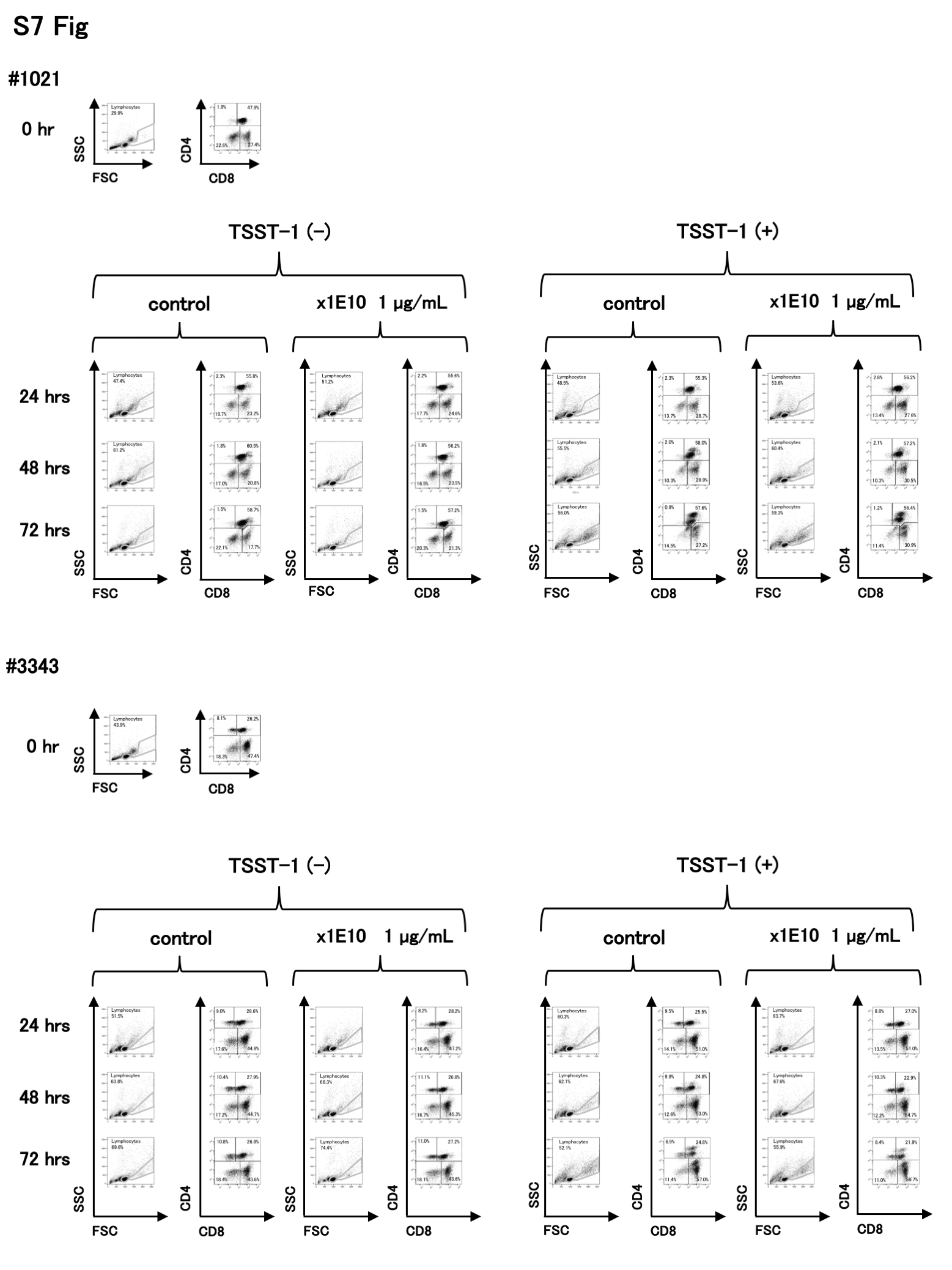

Supplement: S7 Fig — Swine PBMCs (#1021 and #3343) were stimulated in the absence and presence of x1E10. Samples were stained with the mAb (x1E10) followed by anti-mouse IgG-PE. Then, the cells were stained with anti-CD8 mAb and analyzed by FCM as described in Fig 5. Left 4 panels, without TSST-1 stimulation; Right 4 panels, with TSST-1 stimulation. Control groups are stained without x1E10. The x1E10 groups were cultured in the presence of x1E10. The samples were collected at 24, 48 and 72 hrs after the stimulation. The cell size and shape were measured by forward scatter (FSC) and side scatter (SSC). The expressions of CD4 and CD8 were detected in the lymphocyte-gated cells. The data presented in the Figure was used for the Fig 6B. (TIF) [file pone.0242572.s007.tif]
