# Supplementary material for: Preparation and characterization of monoclonal antibodies recognizing two CD4 isotypes of Microminipigs
Source: PLoS One. 2020 Nov 25;15(11):e0242572. doi: 10.1371/journal.pone.0242572 (PMC7688132; doi:10.1371/journal.pone.0242572)
Supplement: S1 Table — (PDF) [file pone.0242572.s008.pdf]

S1_Table.

Gene	Primer name	Primer sequence	References	annealing Temp. (℃)	length (bp)	Allele
**(A) For SLA allele typing by a PCR -SSP method ~~[39][40]~~**						
ACTA1	ACTA1-F	CGCCATGTGTGACGAAGACGAGACC	[39]	65	516	*ACTA1* : porcine α-actin gene (Positive control primer)
	ACTA1-R	CACGTACATGGCGGGCACGTTGAAG	[39]			
SLA-1	SLA1-11F	GTGTCCCGGCCCGACC	[39]	65	183	*11XX (all: 1101/11jh01/11jh02/11mp11/11yn01)
	SLA1-11R	CTGTGCGCTGCCCATGACAC	[39]			
	SLA1-12F	GGTTCGACAGCGACGCCCTC	[39]	65	121	*12XX (all: 1201/12hy01/12Lw01)
	SLA1-12R	CGGTTAATCTGTGCGGTTTCCTTGA	[39]			
	SLA1-13F	GTCTCACACCCTCCAGAGCATGTTT	[39]	65	217	*13XX (all: 1301/13ms21)
	SLA1-13R	CAGTCCCTGCAGGTAGCTCCTCCTA	[39]			
SLA-2	SLA2-04F-3	GAGCCCCGTTTCATCGAAG		60	181	*04XX (all: 0401/040201～02)
	SLA2-04R-3	GCAGGTTCCCTCGGTAAATC				
	SLA2-10F	GCCTCGACACAGAATCTCCGCA	[39]	65	116	*10XX (all: 1001/10an01/10es21/10sk21/10sm01)
	SLA2-10R	CCCGCACTCACCCGCCTGA	[39]			
SLA-3	SLA3-04F	GGAAGCCCCGTTTCATCGAA	[39]	65	192	*04XX (all: 0401 02/04es32)
	SLA3-04R	GCAGGTTTTTCAGGTTCACTCGGA	[39]			
	SLA3-05F	CGTGGAAGATACGCAGTTCGTGT	[39]	65	139	*05XX (all: 0501 03/05sw01)
	SLA3-05R	AGTCTGTGCGTTGTCCTTGCTGA	[39]			
DRB1	DRB1-07F1	GGACCGAGCGGGTGAGGTTCA	[40]	65	133	*07XX (all: 0701/07ka03/07yo02)
	DRB1-07R1	TGGCTGTTCCAGTACTTGGCTGAA	[40]			
	DRB1-10F1	ACGCAGCGCATTTCTTCTTTATGGA	[40]	65	135	*10XX (all: 1001/10jh01/10ka06/10Lu03/10sp07); er01/La03
	DRB1-10R1	GGTACTCGCCCACGTCGCTA	[40]			
DQB1	DQB1-05F1	GCAGCGGGTGCGGCTCT	[40]	65	193	*05XX (all: 0501~03/05sp06)
	DQB1-05R1	TATCTGGTAGTTGTGTTTGCACACC	[40]			
	DQB1-06F1	ACTCAGCGGGTGCGGCA	[40]	65	204	*06XX (all: 0601~02/06sp01); zs12
	DQB1-06R1	GCCTTCCTCTATCTGGTAGTTGTGTTTGC	[40]			
