# Supplementary material for: Preparation and characterization of monoclonal antibodies recognizing two CD4 isotypes of Microminipigs
Source: PLoS One. 2020 Nov 25;15(11):e0242572. doi: 10.1371/journal.pone.0242572 (PMC7688132; doi:10.1371/journal.pone.0242572)
Supplement: S2 Table — (PDF) [file pone.0242572.s009.pdf]

S2_Table.

Gene	Primer name	Primer sequence	References	annealing Temp. (℃)	length (bp)
**(A) For preparation of *CD4.A* and *CD4.B* transfectants**					
CD4	CD4 N53	taGAATTCGGTCTCACCGGTCGCCACCATGGACCCAGGAACCTCTCTGA		58	1421
	CD4 C35	atGATATCGGTCTCGGATCCTCAGGTGAGGGAATAGTTCTTCTG			
	CD4 N53 inner	taGAATTCGGTCTCACCGGTCGCCACCATGGACCCAGGAACCTCTCTGAGGCA		58	1421
	CD4 C35 inner	atGATATCGGTCTCGGATCCTCAGGTGAGGGAATAGTTCTTCTGTTG			
**(B) For CD4 allele typing by a PCR-RFLP method**					
CD4	CD4 exon 3F	CTCAGACTCAAACTGGGATGATTG	[17]	60	366
	CD4 exon 3R	GATCCCAGAGTTTACTAGGAGCTG	[17]		
