# Supplementary material for: Preparation and characterization of monoclonal antibodies recognizing two CD4 isotypes of Microminipigs
Source: PLoS One. 2020 Nov 25;15(11):e0242572. doi: 10.1371/journal.pone.0242572 (PMC7688132; doi:10.1371/journal.pone.0242572)
Supplement: S3 Table — (PDF) [file pone.0242572.s010.pdf]

S3 Table.

swine No.	sex	day of birth	CD4 Type	Haplotype		day of prep		age in month of prep		Fig
						①	②	①	②	
875	♂	2011/9/26	AA	35.23	43.37	2017/10/30		73		3.4
1028	♀	2012/7/19	AA	16.16	43.37	2017/10/30		63		4
1676	♂	2013/9	AA	0.23	0.37	2016/2/16		29		3
1816	♀	2013/11/15	AA	35.23	35.23	2015/3/4	2016/1/21	15	26	2.4
3617	♂	2017/6/16	AA	35.23	35.23	2018/2/19	2018/7/23	8	13	4. 5. 6. S4. S6. S7
1216	♂	2013/3/19	BB	0.11	0.16	2017/10/30		55		3.4
1949	♀	2014/1/31	BB	0.11	0.23	2015/3/4		13		2.4
1932	♀	2014/2	BB	10.11	43.37	2016/2/16		24		3
2858	♂	2015/6/24	BB	35.23	35.23	2019/2/19		43		4. S4. S6. S7
3686	♀	2017/8/10	BB	35.23	35.23	2018/2/19	2018/7/23	6	11	4. 5. 6. S4. S6. S7
3761	♀	2017/10/15	BB	43.37	43.37	2019/7/22		21		5. 6. S4. S6. S7
3762	♀	2017/10/15	BB	43.37	43.37	2019/7/22		21		5. 6. S4. S6. S7
