# Supplementary material for: Preparation and characterization of monoclonal antibodies recognizing two CD4 isotypes of Microminipigs
Source: PLoS One. 2020 Nov 25;15(11):e0242572. doi: 10.1371/journal.pone.0242572 (PMC7688132; doi:10.1371/journal.pone.0242572)
Supplement: S4 Table — (PDF) [file pone.0242572.s011.pdf]

S4 Table.

	antigen	clone	label	company	host	cat No.	final concentration
IHC	swine CD4AB	x1E10			Mouse		100μg/mL
	mouse Ig HRP			NICHIREI	Goat	414171	undiluted solution
	mouse IgG2a			Dako	Mouse	X0943	10μg/mL
FCM	swine CD4AB	x1E10			Mouse		2μg/mL
	swine CD4B	b1D7			Mouse		2μg/mL
	swine MHC class I	X2F6			Mouse		2μg/mL
	swine MHC class I	PT85A		Monoclonal Antibody Center	Mouse	PG-BOV2002	1μg/mL
	swine CD8α	76-2-11	FITC	abcam	Mouse	ab24883	5μg/mL
	mouse IgG	Poly4053	PE	BioLegend	Goat	405307	0.4μg/mL
	mouse IgG1	MGR1-1	APC	BioLegend	Rat	406610	0.2μg/mL
	mouse IgG	Poly4053	APC	BioLegend	Goat	405308	0.4μg/mL

		company	cat No.	final concentration
FCM	streptavidin PE-cy7	BioLegend	405206	0.04μg/mL
