# Supplementary material for: Preparation and characterization of monoclonal antibodies recognizing two CD4 isotypes of Microminipigs
Source: PLoS One. 2020 Nov 25;15(11):e0242572. doi: 10.1371/journal.pone.0242572 (PMC7688132; doi:10.1371/journal.pone.0242572)
Supplement: Attachment — Submitted filename: Response to Reviewers.docx [file pone.0242572.s013.docx]

Date: October 29th 2020

PLOS-ONE Editorial Office

PONE-D-20-14895

**Preparation and characterization of monoclonal antibodies recognizing two CD4 isotypes of Microminipigs**

Dear Editors,

We thank the reviewers for their generous comments on the manuscript and for providing us the opportunity to revise our paper entitled " **Preparation and characterization of monoclonal antibodies recognizing two CD4 isotypes of Microminipigs** " (PONE-D-20-14895).

Your comments and those of the reviewers were highly insightful and enabled us to improve the quality of our English in the manuscript. English of our manuscript was reviewed by the third party. Our point-by-point responses to each of the comments of the reviewer 2 are provided in the ‘revised manuscript with track changes. ’

We hope that the revisions in the manuscript and our accompanying responses will be sufficient to make our manuscript suitable for publication in PLOS ONE.

We look forward to hearing from you at your earliest convenience.

Sincerely,

Name: Yoshie Kametani

Address: Department of Molecular Life science, Division of Basic Medical

Science, Tokai University School of Medicine, Shimokasuya 143,

Isehara-shi, Kanagawa, 259-1193, Japan

E-mail: y-kametn@is.icc.u-tokai.ac.jp

On behalf of all authors,

Reviewer #1: In my opinion this manuscript is fine now. The authors corrected most of the topics. Therer are still some open points but as the authors ,mentioned these can be analyzed in further studies.

Reviewer #2: This revised manuscript has significantly improved writing overall. However, one more improvement in grammar and writing described below will make the manuscript more accessible to readers.

Major comment:
1. Lines 48-56 are about practical applications of developed monoclonal antibodies. Please try to rewrite this paragraph and ask your peers to read with fresh eyes.

We rewrote the lines 48-56 to read as follows:

“Moreover, stimulation of peripheral blood mononuclear cells (PBMCs) derived from CD4.A (+/+) and CD4.B (+/+) swine with toxic shock syndrome toxin-1 (TSST-1) *in vitro* similarly activated both groups of cells that exhibited a slight increase in the CD4/CD8 double positive (DP) cell ratio. A large portion of the DP cells from the allelic CD4.A (+/+) and CD4.B (+/+) groups enhanced the total CD4 and class I swine leukocyte antigen (SLA) expression. The x1E10 mAb delayed and reduced the TSST-1-induced activation of CD4 T cells. Thus, CD4.B appears to be a functional protein whose expression on activated T cells is analogous to CD4.A.”

Minor comments:
2. "Using" would be a more reasonable and concise expression than "By using" in many sentences.

We removed all ‘by’ from ‘by using’.

3. Line 46: Using these two mAbs would be more readable than "By using both of these mAbs".

We corrected according to the reviewer’s suggestion.

4. Line 46: CD4.B allele-specific or unique regions would read better than "CD4.B allele proteins".

We corrected according to the reviewer’s suggestion.
